# Biotechnology and Green Chemistry

**DOI:** 10.1155/2014/590586

**Published:** 2014-03-12

**Authors:** Bernardo Dias Ribeiro, Isabel Marrucho, Luciana Gonçalves, Maria Alice Z. Coelho

**Affiliations:** ^1^Escola de Química, Universidade Federal Rio de Janeiro, 21941-902 Rio de Janeiro, RJ, Brazil; ^2^Instituto de Tecnologia Química e Biológica, Universidade Nova de Lisboa, 2780-157 Oeiras, Portugal; ^3^Departamento de Engenharia Química, Universidade Federal do Ceará, 60455-760 Fortaleza, CE, Brazil

White biotechnology can be regarded as applied biocatalysis, with enzymes and microorganisms, aiming at industrial production of bulk and fine chemicals to food and animal feed additives. In its turn biocatalysis has many attractive features in the context of greenchemistry: mild reaction conditions (physiological pH and temperature); environmentally compatible catalysts and solvent (often water) combined with high activities; and chemo-, regio-, and stereoselectivities in multifunctional molecules. This affords processes which are shorter, generate less waste, and are, therefore, both environmentally and economically more attractive than conventional routes.

This special issue includes aspects involving the use of white biotechnology (enzymes, microorganisms, and plant tissues) within the green chemistry concept, concerning the use of alternative solvents (supercritical fluids, pressurized gases, ionic liquids, and micellar systems) and energies (microwaves and ultrasound); sustainable approaches for production of fine and bulk chemicals (aromas, polymers, pharmaceuticals, and enzymes); use of renewable resources or agroindustrial residues; biocatalysts recycling; and waste minimization.

This special issue contains six papers, where three are related to green synthesis of nanoparticles and one paper covers biological pretreatment for enzymatic hydrolysis and bioethanol production. Two papers regard the use of alternative reaction systems. In the first paper entitled* “Green synthesis of silver nanoparticles using Pinus eldarica bark extract,”* S. Iravani and B. Zolfaghari present the optimization of the biosynthesis production of silver nanoparticles through the evaluation of* Pinus eldarica* bark extract quantity, substrate concentration, temperature, and pH on the formation of such material. The preparation of nanostructured silver particles using* P. eldarica* bark extract provides an environmentally friendly option, as compared to currently available chemical and/or physical methods.

The second paper,* “Green and rapid synthesis of anticancerous silver nanoparticles by Saccharomyces boulardii and insight into mechanism of nanoparticle synthesis,”* by A. Kaler et al. describes an ecofriendly method for the synthesis of silver nanoparticles (AgNPs) by cell free extract (CFE) of* Saccharomyces boulardii*. In addition to the optimization of relevant synthesis parameters as culture age, cell mass concentration, temperature, and reaction time, the paper presents particles characterization by UV-Visible spectroscopy, EDX (energy dispersive X-Rays) analysis, transmission electron microscopy, and zeta potential, as well as the elucidation of proteins/peptides role in nanoparticles formation and stability and their anticancer activity. The method therein described a method that does not require tedious downstream processing and it may be scaled up to develop a viable technology for the Ag-nanoparticle synthesis.

In the third paper,* “Biosynthesis, antimicrobial and cytotoxic effect of silver nanoparticles using a novel Nocardiopsis sp. MBRC-1,”* P. Manivasagan et al. present another green approach for biosynthesis of nanoparticles using the culture supernatant of* Nocardiopsis* sp. MBRC-1 to achieve the reduction of silver ions from a silver nitrate solution. The obtained nanoparticles were characterized by UV-visible, TEM, FE-SEM, EDX, FTIR, and XRD spectroscopy. The prepared silver nanoparticles exhibited strong antimicrobial activity against bacteria and fungi. Cytotoxicity of biosynthesized AgNPs against* in vitro* human cervical cancer cell line (HeLa) showed a dose-response activity.

In the fourth paper,* “Biological pretreatment of rubberwood with Ceriporiopsis subvermispora for Enzymatic hydrolysis and bioethanol production,”* F. Nazarpour et al. investigate a novel feedstock for enzymatic hydrolysis and bioethanol production using biological pretreatment: rubberwood (*Hevea brasiliensis*). To improve ethanol production, rubberwood was pretreated with white rot fungus* Ceriporiopsis subvermispora* to increase fermentation efficiency. The fungal pretreatment provides a cost-effective method for reducing the recalcitrance of rubberwood with high selectivity of lignin degradation rate and minimal cellulose loss for enzymatic hydrolysis and bioethanol production.

In the fifth paper, the research of G. D. Yadav and S. Devendran entitled* “Microwave assisted enzymatic kinetic resolution of (±)-1-phenyl-2-propyn-1-ol in non-aqueous media,”* proposes a kinetic resolution of 1-phenyl-2-propyn-1-ol, an important chiral synthon, through esterification with acyl acetate. The authors investigate synergism between microwave irradiation and enzyme catalysis. The lipase (Novozym 435) catalyzed kinetic resolution under microwave irradiation. The maximum conversion of 48.78% was obtained in 2 h using 10 mg enzyme loading with equimolar concentration of alcohol and ester at 60°C under microwave irradiation. From the progress curve analysis, it was found that reaction followed the ping-pong bi-bi mechanism with dead end inhibition of alcohol. Beside the previous papers herein described, the preparation of chiral secondary alcohols using lipase catalyzed kinetic resolution is mild and clean as compared to chemical process.

In the seventh paper entitled* “Demonstration of redox potential of Metschnikowia koreensis for stereoinversion of secondary alcohols/1,2-diols,”* by V. S. Meena et al. reports the* Metschnikowia koreensis*-catalyzed one-pot deracemization of secondary alcohols/1,2-diols and their derivatives with* in vivo* cofactor regeneration. This ecofriendly method afforded the product in high yield (88%) and excellent optical purity (>98% ee), minimizing the requirement of multistep reaction and expensive cofactor.

*Bernardo Dias Ribeiro* *Isabel Marrucho* *Luciana Gonçalves* *Maria Alice Z. Coelho*

